# The Therapeutic Committee of the British Medical Association

**Published:** 1909-07-15

**Authors:** C. N. Le Brocq


### THE THERAPEUTIC COMMITTEE OF THE BRITISH MEDICAL ASSOCIATION.

BY C. N. LE BROCQ, B.A., M.D.

### Report on the Local Anesthetics recommended as Substitutes for Cocaine.

The substances which have been investigated are: Stovaine, novocaine, tropacocaine, beta-eucaine, alypin, beta-eucaine lactate, nirvanine, holocaine hydrochloride, acoine, orthoform (new) anaesthetine.

The points to which special attention has been paid are those laid down by Professor Braun as essential in estimating a local anaesthetic action. They are:

1. A lower degree of toxicity than cocaine in proportion to its local anaesthetic power.

2. Sufficient solubility in water. The solutions should be stable, that is, they should keep without deterioration and be capable of sterilization by boiling.

3. Absence of any sign of irritation. There should be no injury to the tissues; the local anaesthetic should be easily absorbed without causing any after-effects, such as hyperaemia, inflammation, infiltrations, or necrosis.

4. Compatibility with adrenalin.

5. Rapid penetration of the mucous membrane, and suitability for medullary anaesthesia.

The only exception I would make to these postulates of Braun is that dealing with absorption. It is not obvious that easy absorption is desirable. If the drug is absorbed slowly it remains longer in contact with the nerve fibrils, and produces a more prolonged action. It has been stated that by delaying absorption, as by local vaso-constriction, we are giving the anaesthetic a longer time for action, and it is for this reason that adrenalin is injected with these substances.

If a drug can be obtained which fulfills the above conditions it can be safely said that it will supersede cocaine.

To arrive at a satisfactory conclusion it is necessary to deal with each drug separately, and to discover how closely each approaches the conditions specified by Braun.

### SOLUBILITY IN WATER.

Solubility in water is essential for subcutaneous and intraspinal injections. If a drug is not soluble in water to the extent of forming a 2 per cent. solution, I have regarded it unworthy of competing with cocaine. By examining the solubility of these drugs, the list is considerably diminished, for acoine, holocaine, hydrochloride, anaesthetine, orthoform (new), beta-eucaine, are all more or less insoluble, and for this reason alone unsuitable for producing local anaesthesia by injection. Beta-eucaine is not completely soluble in cold water to the extent of forming a 2 per cent. solution, but if the solution is warmed, a 2 per cent. solution is readily obtained. As dusting powders, these drugs may, of course, still prove useful, but I am not here concerned with that point.

Cocaine, stovaine, novocaine, tropacocaine, beta-eucaine lactate, alypin, and nirvanine are freely soluble in water; their solutions are stable, and as a 2 per cent. solution they will keep a short time without deterioration.

### STERILIZATION OF SOLUTIONS.

Cocaine cannot be boiled, as decomposition occurs, and the drug loses its activity and is gradually destroyed. Stovaine, novocaine, beta-eucaine lactate, tropacocaine, alypin, nirvanine can be sterilized at 115 degs. C. if necessary, undergo no change, and the drug is as active after as before sterilization.

### LOCAL ANAESTHETIC PROPERTIES.

The determination of the local anaesthetic action is not easy, as it is not practicable to work with accuracy on the lower animals on account of the difficulty in determining when sensation is absent or merely blunted. The method I adopted was to take cocaine as the standard and to compare each drug with it separately.

By numerous experiments on frogs, rabbits, and human beings, which will be described in detail elsewhere, I arrived at the following conclusions:

Stovaine has a more powerful anaesthetic action, weight for weight, than any of the other local anaesthetics. Alypin, beta-eucaine lactate, novocain, and tropacocaine have anaesthetic properties about equal to cocaine. Nirvanine as a local anaesthetic is inferior to cocaine.

### TOXICITY.

Having determined the relative anaesthetic action, it remained to estimate the toxicity of these drugs. The method employed was to find the minimal lethal dose—the smallest dose which will kill the animal—in frogs, mice, and rabbits.

All these drugs produce death by paralyzing the central nervous system in mammals and the heart in frogs. When a toxic dose of a drug is injected into a frog, movements gradually cease, the animal soon lies still in any position in which it is placed, and becomes to all external appearance dead; respiration ceases, and there is no response to any sort of stimulus. If the dose has not been large enough to paralyze the heart, it continues to beat, and as soon as the effects of the drug on the nervous system pass off the frog recovers. The explanation of the recovery is, of course, that a frog can exist for a considerable time, even for many hours, without respiration. Thus in obtaining the minimal lethal dose in frogs it is necessary to give these drugs in doses which not only paralyze the nervous system, but which are large enough to paralyze the heart also.

Therefore the minimal lethal dose in frogs represents the action of the drug on the heart, and in mammals its action on the central nervous system.

### TOXICITY IN FROGS.

The minimal lethal dose for a 20 gram frog, using a 2 per cent solution, is for:

| | | |
|---|---|---|
| Alypin | 6 minims | (most toxic) |
| Cocaine | 12 " | |
| Stovaine | 15 " | |
| Nirvanine | 17 " | |
| Beta-eucaine lactate | 20 " | |
| Tropacocaine | 20 " | |
| Novocaine | 40 " | (least toxic) |

### TOXICITY IN MICE.

The minimal lethal dose for a 20-gram mouse using a 2 per cent, solution, is for:

| | |
|---|---|
| Alypin | 4 minims. |
| Cocaine | 5 " |
| Nirvanine | 7 " |
| Stovaine | 8 " |
| Novocaine | 10 " |
| Tropacocaine | 10 " |
| ⅛eta-eucaine lactate | 12 " |

### TOXICITY IN RABBITS.

The minimal lethal dose for a rabbit weighing 1,000 grams using a 10 per cent. solution, is for:

| | | | | | |
|---|---|---|---|---|---|
| Alypin | between | 18 | and | 23 | minims. |
| Cocaine | " | 24 | " | 30 | " |
| Stovaine | " | 35 | " | 45 | " |
| Tropacocaine | " | 44 | " | 54 | " |
| Novocaine | " | 53 | " | 63 | " |
| Beta-eucaine lactate | " | 65 | " | 75 | " |

As the toxic action of these drugs is to paralyze the nervous system, and so the respiratory centre, the toxicity in mammals must be regarded as the correct reading, for when respiration ceases the animal dies.

If the toxicity of Cocaine be represented as 1, then

| | | | | | |
|---|---|---|---|---|---|
| The toxicity | of Alypin | will | represent | 1.25 |
| " | " Cocaine | " | " | 1.0 |
| " | " Nirvanine | " | " | 0.714 |
| " | " Stovaine | " | " | 0.625 |
| " | " Tropacocaine | " | " | 0.500 |
| " | " Novocaine | " | " | 0.490 |
| " | " Beta-eucaine lactate | " | " | 0.414 |

### CONCLUSIONS FROM THE ABOVE.

Alypin has anaesthetic powers equal to cocaine, but a higher toxicity, and so does not comply with the conditions enunciated by Braun. Nirvanine has not the anaesthetic power of cocaine, and it is only slightly less toxic. As. however, we have four drugs equal to or stronger than cocaine in anaesthetic power, and considerably less toxic than nirvanine, no further experiments with this drug were deemed necessary.

From this it will be seen that only four drugs, namely, stovaine, novocaine, tropococaine, and beta-eucaine lactate have complied with the first two conditions. With these four drugs further experiments were performed to discover how they fulfill the other conditions laid down for the "perfect local anaesthetic."

### IRRITANT ACTION ON THE TISSUES.

Very little is known of the action of the local anaesthetics on the tissues, with which they come in contact. This effect is, however, extremely important, for it has been stated that gangrene and sloughing of the tissues have followed the use of certain of these drugs. It is well recognized, however, that they are all general protoplasmic poisons, that whilst having a special predilection for nervous structures they depress and ultimately destroy every form of living tissue.

In these, as in previous experiments, cocaine was taken as the standard. Cocaine is generally recognized as having a slight irritant action on the tissues, and occasionally, after instillation into the eye, produces considerable conjunctivitis.

For these experiments rabbits weighing about 1 kilogram were used. The abdomen was shaved and the skin washed and made aseptic, after which 10 minims of a 10 per cent. solution of the drug was injected subcutaneously. The drug was carefully sterilized, and all antiseptic precautions were observed.

After injection the part was kept under immediate observation for several hours, and then inspected daily for one week more.

Cocaine caused slight swelling and hyperaemia soon after the injection. The part completely recovered.

Stovaine caused intense hyperaemia and dilitation of the blood vessels, followed by sloughing of the part.

Beta-eucaine lactate caused swelling and thickening about the seat of injection, followed by sloughing.

Tropacocaine caused swelling and some thickening, followed by sloughing.

Novococaine showed no swelling and no hyperaemia. The part was perfectly normal after injection, and remained so.

In the above experiments, where sloughing and necrosis occurred there were no signs of pus formation.

It is seen that a comparative investigation into the action of these drugs on the general tissue is of the utmost importance, for most of them possess a decided irritant effect; so much so, that they will produce sloughing and necrosis of the tissues with which they come in contact.

A 10 per cent. solution is a stronger solution than is generally used therapeutically, yet this strength is still employed; moreover, 10 minims of a 10 per cent. solution roughly represents 1 grain, a quantity which is far exceeded when weaker solutions are injected. And even if it be objected that it is not fair to infer from a 10 per cent. solution what will occur in a strength of 1 per cent. or 2 per cent., yet the present experiments still show the relative irritant action of these drugs. A drug which is only mildly irritant as a 1 per cent. solution, will presumably be the more irritant as a 2 per cent. solution, until, as the strength is increased, a stage is reached when destruction of the tissues and necrosis is obtained.

The irritant action of stovaine, beta-eucaine lactate and tropacocaine is far greater than that of cocaine; novocain the only drug which is superior to cocaine in this respect.

### COMPATABILITY WITH ADRENALIN.

All the local anaesthetics are compatible with adrenalin if the solutions are fresh and kept only for a short time. After a day or two the adrenalin decomposes unless it is kept in stoppered opaque bottles.

### CONCLUSIONS.

In determining which of these four drugs is the most suitable substitute for cocaine, it is necessary to compare them with one another.

If novocaine and tropacocaine be first compared, their toxicity and anaesthetic properties are, roughly, equal; but the irritant action of tropacocaine is far greater than that of novococaine; in other respects their actions are similar, therefore novocain is a more suitable drug than tropacocaine.

On comparing novocain with beta-eucaine lactate it is seen that while the anaesthetic value is roughly about equal the toxicity of beta-eucaine lactate is slightly less than that of novocain, but the irritant action of beta-eucaine lactate is far greater than that of novocaine. It appears, then, that while beta-eucaine lactate has only a slighter degree of toxicity to recommend it in preference to novocaine, its irritant action far and away overshadows any such slight advantage, and novocain is recognized as undoubtedly the better drug of the two.

Finally, it only remains to compare novocaine with stovaine. The former drug is less toxic and much less irritant; indeed, its specific action on nerve fibres is so great that it has practically no destructive effect on the other tissues; stovaine is more toxic and considerably more irritant.

The one definite advantage which stovaine possesses over all the other local anaesthetics is its greater injurious action on nerve fibres, as shown by anaesthesia. Nevertheless the specific action of stovaine on nerve fibres is less than that of novocaine, since stovaine destroys other tissues besides nerve fibres. If stovaine and novocaine be given in doses so that their anaesthetic action is the same, both the irritant and toxic effect of the former drug, even in the smaller dose in which it is administered, are greater than the relatively larger doses of the latter.

I come to the conclusion, therefore, that of the drugs which have been investigated, novocaine is most satisfactory for general use. Its anaesthetic action is equal to that of cocaine, and its toxicity and general destructive power on the tissues are very much less.—*British Journal of Dental Science.*

